# 

**Published:** 1885-05

**Authors:** 


CONTENTS.

Vol. 32 No. 5

| | PAGE. |
|---|---|
| Preserved Sunshine | 1 |
| Clean your Cellars | 2 |
| Sleeplessness | 3 |
| The First Signs of Consumption, | 4 |
| Measurements of the Depth of Sleep | 5 |
| Writer's Cramp Cured | 5 |
| Porpoise Flesh as Food | 6 |
| Light in the Sick Room | 7 |
| Going without a Religion | 7 |
| On Life Saving from Drowning by Self Inflation | 9 |
| The Use of Iodoform | 10 |
| Kumiss Kefir | 10 |
| Anglo-Swiss Milk Food | 12 |
| Facial Neuralgia Treated by Nerve Vibration | 13 |
| Dentition | 14 |
| Chiromancy | 14 |
| Brain Diseases | 15 |
| Poisons and Antidotes | 15 |
| Book Notices | 17 |
| The Finding of Greely | 17 |
| Unhealthy Bread | 18 |
| Notes and Miscellany | 19 |

